# Supplementary material for: Anharmonic strong-coupling effects at the origin of the charge density wave in CsV3Sb5
Source: Nat Commun. 2024 Mar 1;15:1895. doi: 10.1038/s41467-024-45865-0 (PMC10907679; doi:10.1038/s41467-024-45865-0)
Supplement: Supplementary file 1 — Supplementary Information [file 41467_2024_45865_MOESM1_ESM.pdf]

Supplementary Materials
Anharmonic Strong-Coupling Effects at the Origin of the Charge Density Wave in CsV_3Sb_5

Ge He[1,2,12]✉, Leander Peis[1,3,4,10,12], Emma Frances Cuddy[5,6,12], Zhen Zhao[7], Dong Li[7], Yuhang Zhang[7], Romona Stumberger[1,3,11], Brian Moritz[6], Haitao Yang[7,8]✉, Hongjun Gao[7,8], Thomas Peter Devereaux[5,6,9]✉ and Rudi Hackl[1,3,4]✉

[1] *Walther Meissner Institut, Bayerische Akademie der Wissenschaften, Garching 85748, Germany*

[2] *Department of Physics, University College Cork, College Road, Cork T12 K8AF, Ireland*

[3] *School of Natural Sciences, Technische Universität München, Garching 85748, Germany*

[4] *IFW Dresden, Helmholtzstrasse 20, Dresden 01069, Germany*

[5] *Department of Materials Science and Engineering, Stanford University, Stanford, California 94305, USA*

[6] *Stanford Institute for Materials and Energy Sciences, SLAC National Accelerator Laboratory and Stanford University, 2575 Sand Hill Road, Menlo Park, California 94025, USA*

[7] *Beijing National Laboratory for Condensed Matter Physics, Institute of Physics, Chinese Academy of Sciences, Beijing 100190, China*

[8] *School of Physical Sciences, University of Chinese Academy of Sciences, Beijing 100049, China*

[9] *Geballe Laboratory for Advanced Materials, Stanford University, Stanford, California 94305, USA*

[10] *Present address: Capgemini, Frankfurter Ring 81, 80807 München, Germany*

[11] *Present address: Robert Bosch GmbH, Robert-Bosch-Campus 1, 71272 Renningen, Germany*

[12] *These authors contributed equally: Ge He, Leander Peis, Emma Frances Cuddy.*

✉ *e-mail: ghe@ucc.ie; htyang@iphy.ac.cn; tpd@stanford.edu; Hackl@tum.de*

A. Sample quality

XRD pattern was collected using a Rigaku SmartLab SE X-ray diffractometer with Cu Kα radiation ($\lambda = 0.15418$ nm) at room temperature. Figure 1 **a** shows the XRD characterizations of the CsV_3Sb_5 single crystal, which exhibits a single preferential orientation of $(00l)$ as marked in the figure. The magnetic susceptibility was determined by a SQUID magnetometer (Quantum Design MPMS XL-1). The CDW transition was estimated to occur at 95 K from the sharp drop in the magnetization curve under an external magnetic field of 1 T, as seen in Fig. 1 **b**. In-plane electrical resistance and its first derivative data were collected on a Quantum Design Physical Properties Measurement System (PPMS), where the CDW transition can be clearly identified as a kink in the RT plot (see Fig. 1 **c**) and a dip-hump feature in the dR/dT plot (see Fig. 1 **d**).

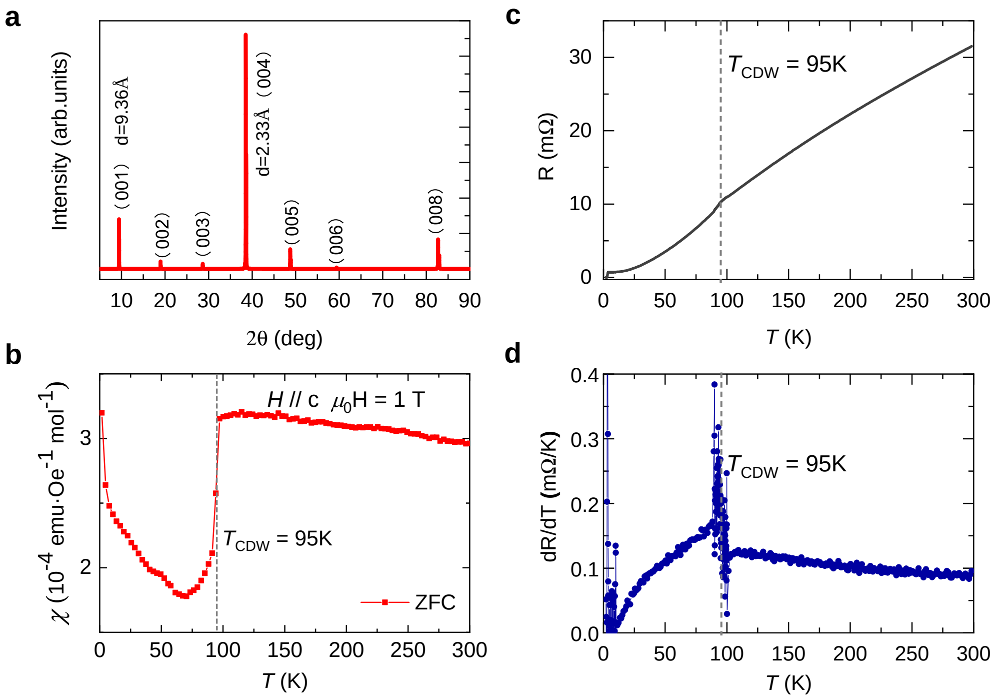

Figure 1. **Characterization of single-crystalline CsV_3Sb_5.** **a** The $\theta - 2\theta$ scan shows only $(00l)$ peaks. **b** Temperature dependence of magnetization. The field is parallel to *c*-axis of the sample. The CDW transition occurs at 95 K. **c** and **d** In-plane electrical resistance and its first derivative as a function of temperature.

B. Crystal harmonic functions

Based on the Raman selection rules, RR and RL configurations project out the excitations with $A_{1\mathrm{g}}$ and $E_{2\mathrm{g}}$ symmetry, respectively. The crystal harmonics of $A_{1\mathrm{g}}$ and $E_{2\mathrm{g}}$ symmetry, to which Raman vertices are proportional, can be derived using the method described in Ref. [1]. The vertices read as follows:

$$\begin{cases} \Phi_{A_{1g}^{1st}}(\boldsymbol{k}) = \dfrac{1}{3}\left[\cos(k_x a) + 2\cos\left(\dfrac{1}{2}k_x a\right)\cos\left(\dfrac{\sqrt{3}}{2}k_y a\right)\right] \\ \Phi_{A_{1g}^{2nd}}(\boldsymbol{k}) = \dfrac{1}{3}\left[\cos(2k_x a) + 2\cos\left(k_x a\right)\cos\left(\sqrt{3}k_y a\right)\right] \\ \Phi_{E_{2g}^{1}}(\boldsymbol{k}) = \dfrac{2}{3}\left[\cos(k_x a) - \cos\left(\dfrac{1}{2}k_x a\right)\cos\left(\dfrac{\sqrt{3}}{2}k_y a\right)\right] \\ \Phi_{E_{2g}^{2}}(\boldsymbol{k}) = 2\sin\left(\dfrac{1}{2}k_x a\right)\sin\left(\dfrac{\sqrt{3}}{2}k_y a\right) \end{cases} \tag{1}$$

where a is the in-plane lattice constant.

C. CDW gap excitation with different laser lines

Raman spectra in both A_{1g} and E_{2g} symmetry, excited by the laser lines at 575 nm, 514 nm and 476 nm are shown in Fig. 2. We find similar gaps for all laser lines, exhibiting intersection points at around 1400 cm^{-1} for both symmetries. This agreement supports the Raman scattering origin of the excitations and the absence of strong resonance effects. There may be some extra contribution from luminescence in the E_{1g} spectra measured at 476 nm [panel **f**].

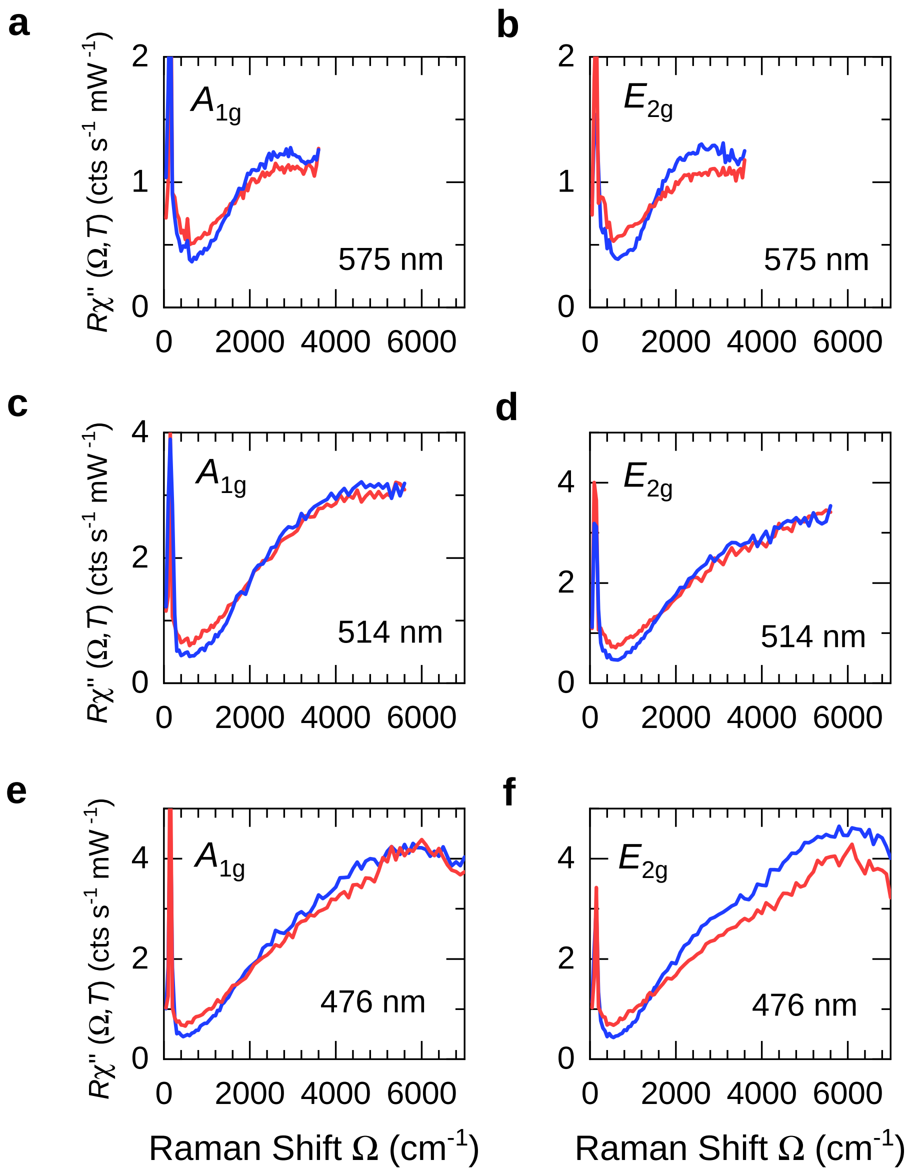

Figure 2. Spectra excited by yellow ($\lambda_L = 575$ nm), green ($\lambda_L = 514$ nm) and blue laser light ($\lambda_L = 476$ nm) in A_{1g} and E_{2g} symmetries are shown in **a**, **c**, and **e** and in **b**, **d**, and **f**, respectively.

D. Low energy spectra for different excitation lines

The low energy Raman spectra in both A_{1g} and E_{2g} symmetry for the blue (476 nm), green (514 nm) and yellow (575 nm) laser excitation, are shown in Fig. 3. The emerging peaks below T_{CDW} show weak dependencies of the positions on the excitation energies for reasons we do not know. The strongest variation is observed for the A_{1g} lines at 40-45 cm^{-1} and around 100 cm^{-1}. They may explain why sometimes two lines were observed.

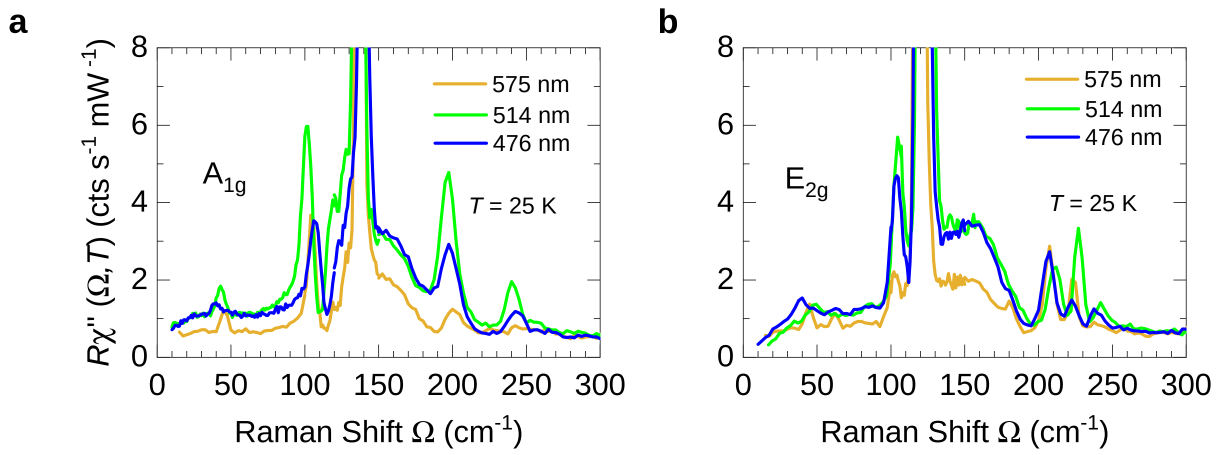

Figure 3. **Low energy Raman spectra as a function of excitation line.** **a** A_{1g} symmetry. **b** E_{2g} symmetry.

E. Fits to the zone-folded (ZF) phonons and amplitude modes (AMs) in A_{1g} and E_{2g} symmetries

To perform a quantitative analysis of the AMs and ZF phonons in A_{1g} and E_{2g} symmetries, we utilize Voigt functions to fit these peaks. As a special case, the AM in A_{1g} spectra shows an asymmetric shape, typically resulting from the interaction between continuous states and a discrete state, as proposed by Fano [2]. To fit the A_{1g} AM, we use a Gaussian convoluted Fano function [3], described in more detail in the section H. The Gaussian width corresponds to the spectral resolution and is set at $4.3\,\mathrm{cm}^{-1}$ in this work. We present the fits to these lines in Fig. 4 **a** and **b**.

The fitting parameters, which include peak energies and linewidths as a function of temperature, are displayed in Fig. 4 **c** and **d**. By examining the temperature-dependent peak energies and linewidths, we can differentiate between AMs and ZF phonons. Typically, the temperature dependence of phonons is weak below 100 K, and changes of less than 5% [4]. In contrast, the AM experiences a significant decrease as T approaches the phase transition temperature [5, 6]. In comparison to the phonons, the peaks at around $105\,\mathrm{cm}^{-1}$ and $208\,\mathrm{cm}^{-1}$ in the A_{1g} and in E_{2g} spectra, respectively, exhibit strong temperature dependencies in both energy and linewidth. Consequently, we identify these two peaks as AMs instead of ZF phonons.

The phonon linewidths are fitted using an anharmonic symmetric decay model [7]:

$$\Gamma_i(T) = \Gamma_{i,0}\left[1 + \frac{2\lambda_{i,\mathrm{ph-ph}}}{\exp\left(\frac{\hbar\omega_0}{2k_BT}\right) - 1}\right] \tag{2}$$

where $\lambda_{i,\mathrm{ph-ph}}$ is the phonon-phonon coupling constant, and ω_0 is the corresponding phonon energy in the ground state. The phonon-phonon coupling constants are listed in Tab. I. They range between 0.37 and 1.11.

The phonon energy shift depends on both the anharmonic decay and the thermal expansion. With taking both into account, the temperature dependence of the phonon energy $\omega_i(T)$ can be given by [8]:

$$\omega_i(T) = \omega_{i,0} + \omega_{i,0}\left\{\exp\left[-3\gamma_i\int_0^T \alpha_V(T')dT'\right] - 1 - \left(\frac{\Gamma_{i,0}}{\sqrt{2}\omega_{i,0}}\right)^2\left[1 + \frac{4\lambda_{i,\mathrm{ph-ph}}}{\exp\left(\frac{\hbar\omega_{i,0}}{2k_BT}\right) - 1}\right]\right\} \tag{3}$$

where γ_i is the Grüneisen parameter of the mode i, and $\alpha_V(T')$ is the coefficient of volume thermal expansion at temperature T'.

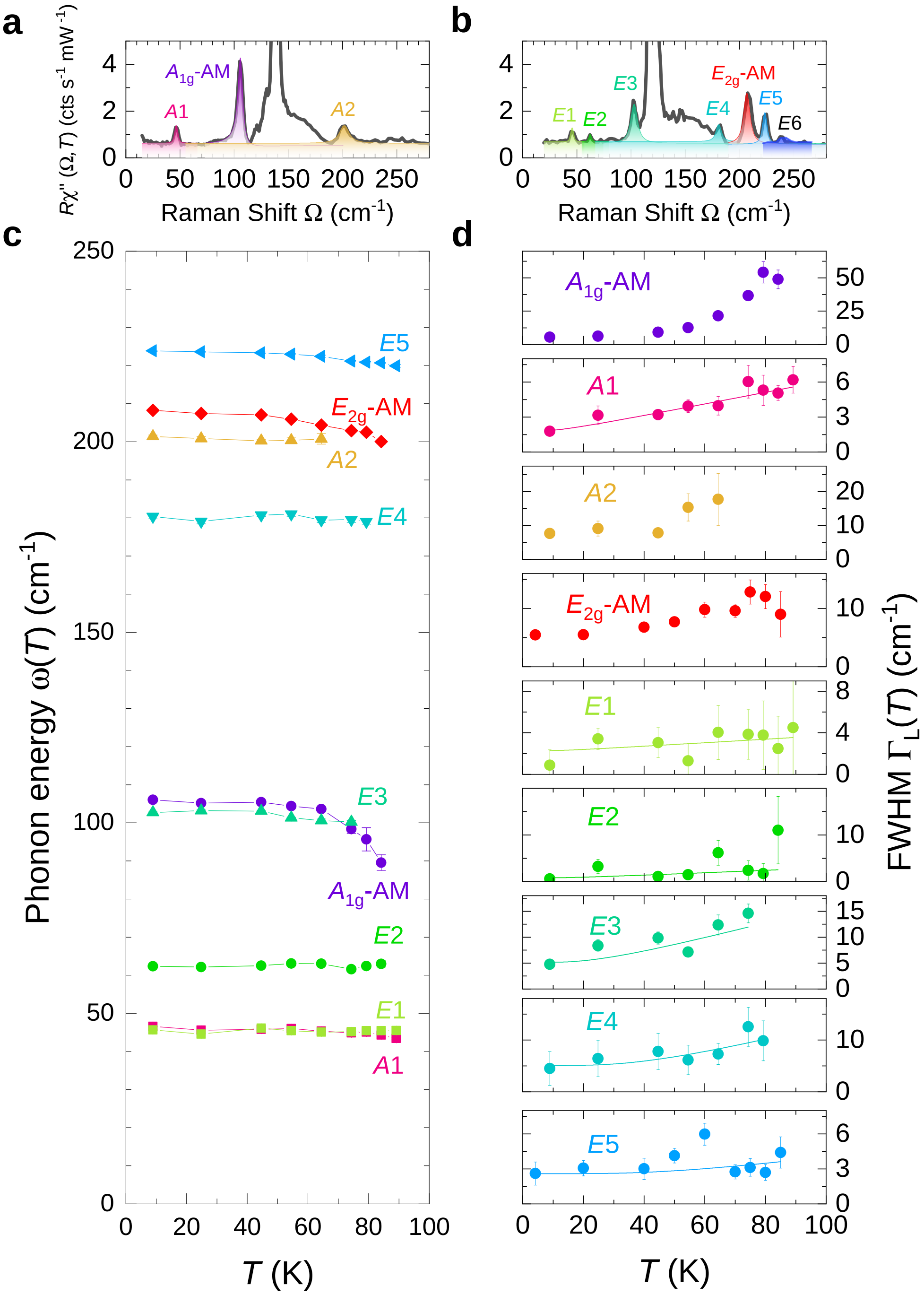

Figure 4. **Temperature dependence of energies and linewidths of AMs and ZF phonons.** **a** and **b** Fits of ZF phonons and AMs in A_{1g} and E_{2g} symmetries. **c** Line energies in A_{1g} and E_{2g} spectra as a function of temperature. **d** The corresponding linewidths of these peaks were fitted with an anharmonic symmetric decay model [7]. Peaks A2 and E6 are not included, since the intensity is too low.

Phonon	A1	E1	E2	E3	E4	E5
$\lambda_{\text{ph-ph}}$	0.44 ± 0.08	0.37 ± 0.2	0.72 ± 1.31	1.09 ± 0.58	0.95 ± 0.76	1.11 ± 0.74

Table I. Phonon-phonon coupling parameters derived from the temperature dependence of the linewidths in Fig. 4 **d**. Below the transition, there are three Raman active phonons in the A_{1g} and seven Raman active phonons in the E_{2g} channel, respectively.

F. Fluctuations

CDW fluctuations may induce extra contributions slightly above T_{CDW} in the electronic Raman spectra, superimposed on the continuum, as observed in $ErTe_3$ [5]. However, this is not the case in CsV_3Sb_5, where the measured spectra show no comparable extra contributions in both A_{1g} and E_{2g} symmetries, as shown in Fig. 5. Apparently, CDW fluctuations cannot be observed in CsV_3Sb_5. Since fluctuations in systems with a large gap ratio are observed only rarely [5] they cannot safely be excluded here but remain as a possibility for explaining the gap ratio.

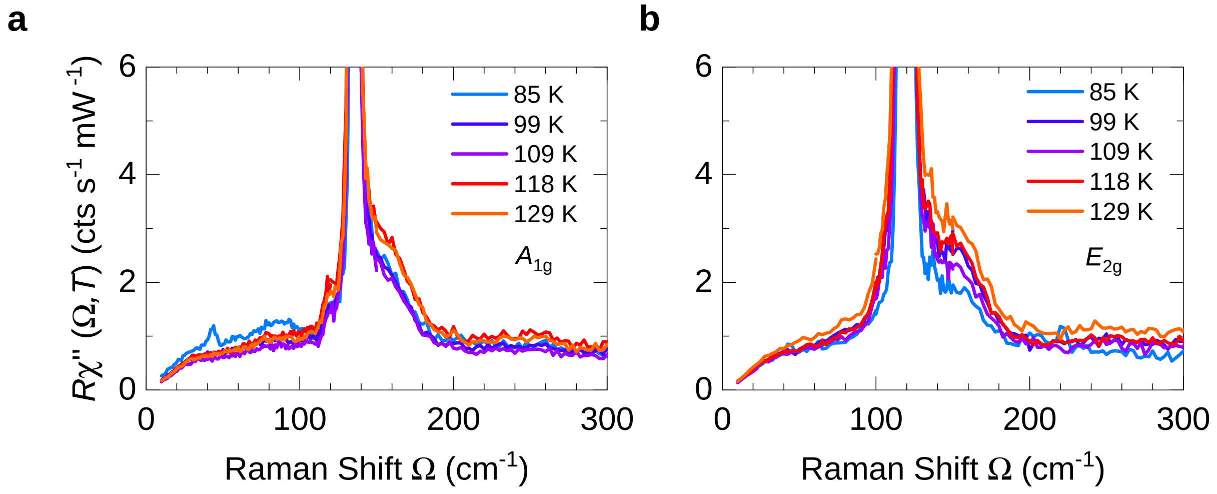

Figure 5. **Raman spectra in A_{1g} and E_{2g} symmetries at temperatures around T_{CDW}. a** A_{1g} symmetry. **b** E_{2g} symmetry. Fluctuations are expected to appear at energies of order temperature as additional structure above 100 K. Neither in A_{1g} nor E_{2g} additional peaks could be observed in CsV_3Sb_5. For various reasons, however, this absence is not conclusive for discarding fluctuations.

G. Band structure and phonon properties in pristine-, SoD- and iSoD-distorted-lattice of CsV_3Sb_5 .

Figure 6 shows the band structure and density of states in pristine-, SoD- and iSoD-distorted-lattice of CsV_3Sb_5 . These calculations were conducted using the folded Brillouin Zone of the 2×2 distortion. Splitting between the pristine and SoD- and iSoD-distorted-lattices shows the large gap at M (green arrow in Fig. 6 **a**), and smaller gaps on the $K - \Gamma$ path (red arrows in Fig. 6 **a**). The phonon frequencies of the A_{1g} phonon in three different lattices are presented in Table II. This finding is consistent with our experimental observations, where the energy of the A_{1g} phonon jumps from 136.0 cm^{-1} to 137.3 cm^{-1} across T_{CDW} upon cooling. In contrast, no such jump was observed in the E_{2g} mode due to less mode consistency in the distorted state. Investigations into electron phonon coupling of the A_{1g} and E_{2g} modes indicated negligible coupling between these modes and the electronic continuum, as expected from the lineshapes of the experimental Raman data.

Mode	Pristine	SoD	iSoD	Exp.(T>T_{CDW})	Exp.(T<T_{CDW})
A_{1g}	141.0	141.6	141.6	136.0	137.3

Table II. Comparison of phonon energy (measured in cm^{-1}) between calculations and Raman experiments (Data are taken near T_{CDW}.).

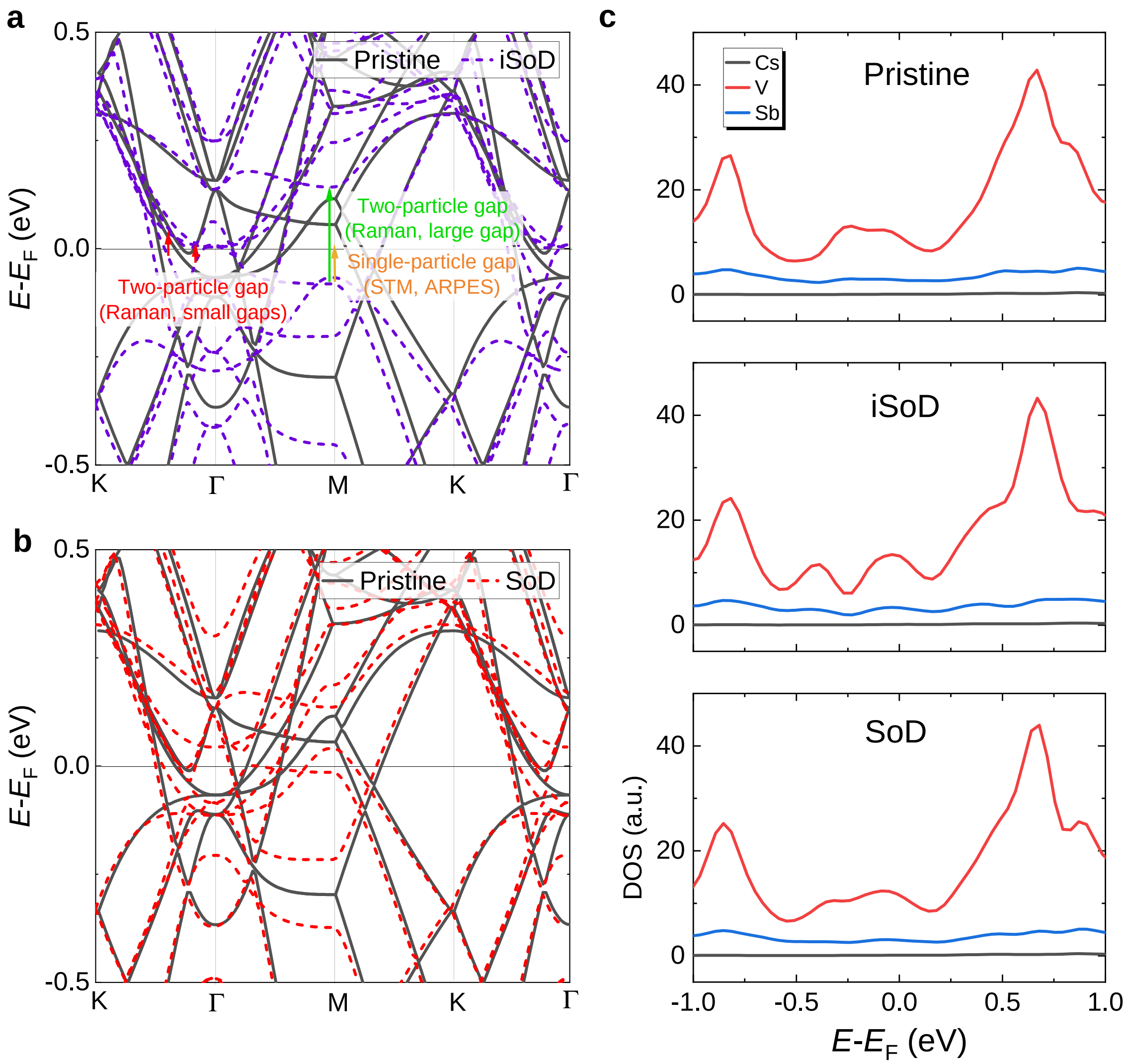

Figure 6. **Band structure and density of states for pristine-, SoD- and iSoD-distorted- lattice as indicated.** **a** and **b** Band structures. The variation with distortion is of order 100 meV or less. The small and large gaps captured by the calculations of the joint density state are indicated with red (along Γ-K line) and green arrows (at M point), respectively. For comparison, the single-particle excitation gap is also illustrated with orange arrow at M point. **c** Density of states (DOS). Changes around the Fermi energy are relevant for the position of the phonons.

H. Asymmetric Amplitude Mode

The observable asymmetric shape of the mode in A_{1g} at $\approx 105\,\mathrm{cm}^{-1}$, along with the previously mentioned significant changes in energy and linewidth, leads to its identification as an AM. To account for the unconventional shape, we compared the fitting of this peak using a Fano function and two Lorentzian peaks. All functions were convoluted with a fixed Gaussian to account for the resolution of the spectrometer, as mentioned in Section E. Prior to the fitting process, we carefully subtracted the background continuum using the Drude Lorentzian form [9] (indicated by the grey lines in Fig. 7). Both the Fano and two Voigt (Lorentzian convoluted with Gaussian) fittings match quite well as seen in Fig. 7. However, the energy and linewidth of the two Lorentzian peaks do not exhibit a dependence on temperature as would be expected from phononic modes or an AM. This is likely due to overfitting of these two peaks, as shown in Fig. 8 **a**. In contrast, the temperature dependent energy and linewidth of the Fano peak seems more reasonable (see Fig. 8 **b**). The corresponding R^2-values are presented in Fig. 8 **c**. Based on the R^2 values, although the two Lorentzian peaks come close, it is evident that the data is better represented by a Fano function. By describing this peak with a Fano function, we can extract the asymmetry parameter $1/|q|$, which is shown in the main text of Fig. 4.

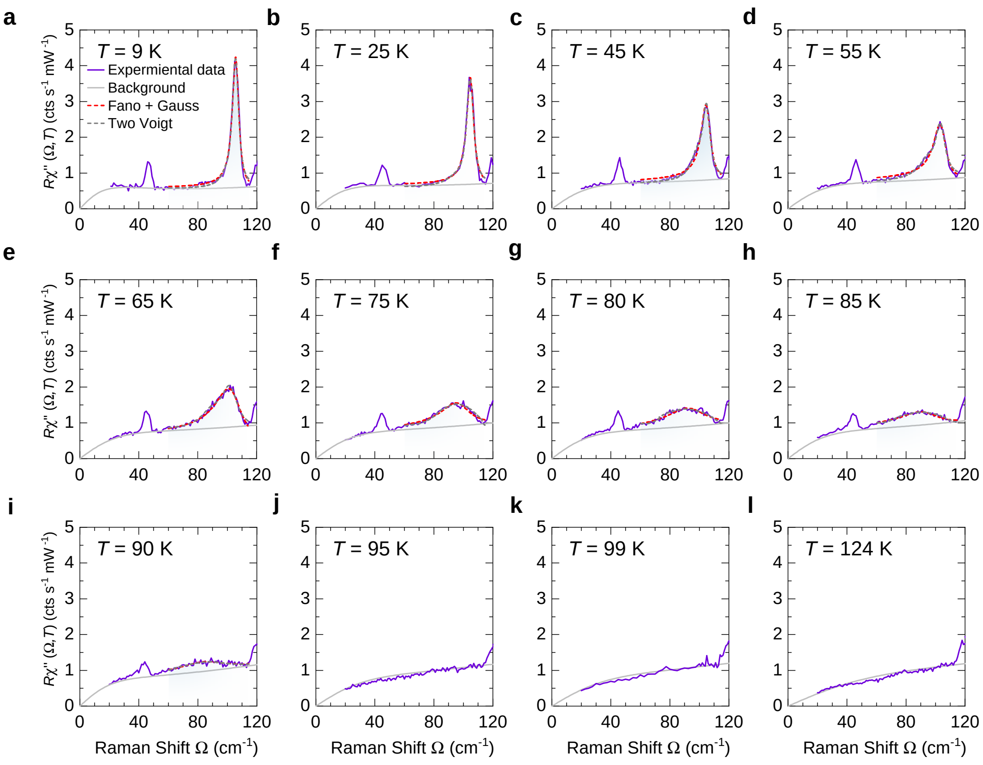

Figure 7. **Fitting results of the A_{1g}-AM comparing different peak shapes. a-l** Raman spectra and their fitting results at different temperatures as indicated. The grey line represents a Drude-Lorentz description of the data borrowed fron the IR analysis. The generic lines are convoluted with a Gaussian to account for the resolution.

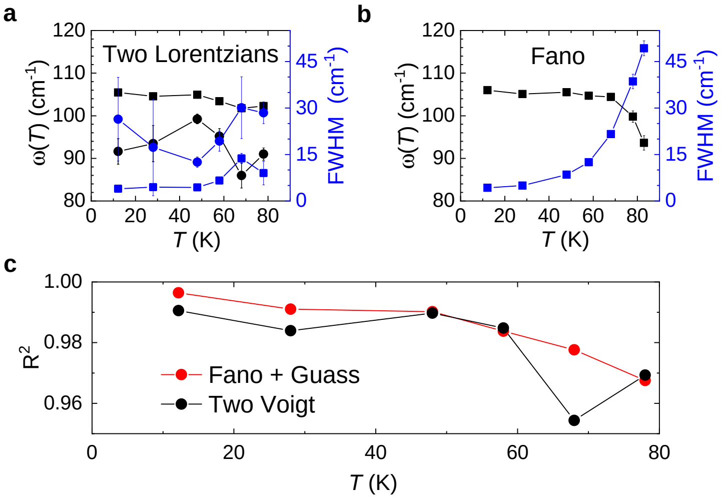

Figure 8. **Fitting parameters of the A_{1g}-AM comparing different peak shapes. a** and **b** Temperature dependencies of the peak positions and linewidths for the Fano **a** and the two Lorentzians **b**. **c** The R^2 value for the Fano+Gauss- and two-Voigt-fits.

I. AM in other well-known CDW materials

For a simple comparison of the peak shape of the observed AM in CsV_3Sb_5 with several well-studied AM modes, published data were extracted presented in Fig. 9. The chosen compounds for comparison were 2H-$TaSe_2$[10], $K_{0.3}MoO_3$ [11], $LaTe_3$ [12] and $ErTe_3$ [5], as they exhibit distinct AM peaks. In all cases, the AM lines can be fitted using Lorentzian profiles convoluted with a Gaussian for the instrumental resolution, indicating symmetric AM lines in these compounds. The temperature dependence of the peak frequencies are compiled in Fig. 5 **d** of the main text.

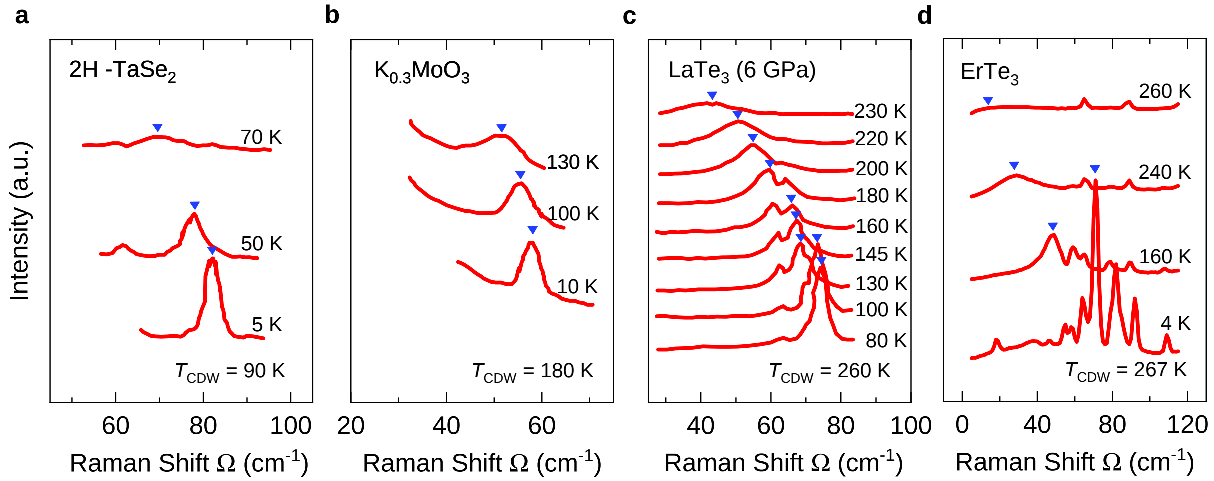

Figure 9. **Experimental results for AMs in various coumpounds.** The AMs are marked with blue triangles. **a** 2H-$TaSe_2$ [10], **b** $K_{0.3}MoO_3$ [11], **c** $LaTe_3$ [12] and **d** $ErTe_3$ [5].

REFERENCES

[1] S. Djurdjić Mijin, A. Baum, J. Bekaert, A. Šolajić, J. Pešić, Y. Liu, G. He, M. V. Milošević, C. Petrovic, Z. V. Popović, R. Hackl, and N. Lazarević, Probing charge density wave phases and the mott transition in $1t - tas_2$ by inelastic light scattering, Phys. Rev. B **103**, 245133 (2021).

[2] U. Fano, Effects of Configuration Interaction on Intensities and Phase Shifts, Phys. Rev. **124**, 1866 (1961).

[3] S. Schippers, Analytical expression for the convolution of a fano line profile with a gaussian, J. Quant. Spec. Radial. Trans. **219**, 33 (2018).

[4] G. He, L. Peis, R. Stumberger, L. Prodan, V. Tsurkan, N. Unglert, L. Chioncel, I. Kézsmárki, and

R. Hackl, Phonon anomalies associated with spin reorientation in the kagome ferromagnet fe3sn2, Phys. Status Solidi B **259**, 2100169 (2022).

[5] H.-M. Eiter, M. Lavagnini, R. Hackl, E. A. Nowadnick, A. F. Kemper, T. P. Devereaux, J.-H. Chu, J. G. Analytis, I. R. Fisher, and L. Degiorgi, Alternative route to charge density wave formation in multiband systems, Proc. Natl. Acad. Sci. **110**, 64 (2013).

[6] R. Grasset, Y. Gallais, A. Sacuto, M. Cazayous, S. Mañas Valero, E. Coronado, and M.-A. Méasson, Pressure-induced collapse of the charge density wave and higgs mode visibility in $2h{-}\text{tas}_2$, Phys. Rev. Lett. **122**, 127001 (2019).

[7] P. G. Klemens, Anharmonic decay of optical phonons, Phys. Rev. **148**, 845 (1966).

[8] H.-M. Eiter, P. Jaschke, R. Hackl, A. Bauer, M. Gangl, and C. Pfleiderer, Raman study of the temperature and magnetic-field dependence of the electronic and lattice properties of MnSi, Phys. Rev. B **90**, 024411 (2014).

[9] T. P. Devereaux and R. Hackl, Inelastic light scattering from correlated electrons, Rev. Mod. Phys. **79**, 175 (2007).

[10] H. M. Hill, S. Chowdhury, J. R. Simpson, A. F. Rigosi, D. B. Newell, H. Berger, F. Tavazza, and A. R. Hight Walker, Phonon origin and lattice evolution in charge density wave states, Phys. Rev. B **99**, 174110 (2019).

[11] G. Travaglini, I. Mörke, and P. Wachter, Cdw evidence in one-dimensional $K_{0.3}MoO_3$ by means of Raman scattering, Solid State Commun. **45**, 289 (1983).

[12] M. Lavagnini, H.-M. Eiter, L. Tassini, B. Muschler, R. Hackl, R. Monnier, J.-H. Chu, I. R. Fisher, and L. Degiorgi, Raman scattering evidence for a cascade evolution of the charge-density-wave collective amplitude mode, Phys. Rev. B **81**, 081101 (2010).
