# Supplementary material for: Anharmonic strong-coupling effects at the origin of the charge density wave in CsV3Sb5
Source: Nat Commun. 2024 Mar 1;15:1895. doi: 10.1038/s41467-024-45865-0 (PMC10907679; doi:10.1038/s41467-024-45865-0)
Supplement: Supplementary file 4 — Lasing Reporting Summary [file 41467_2024_45865_MOESM4_ESM.pdf]

nature research

Corresponding author(s): Ge He

Lasing Reporting Summary

Nature Research wishes to improve the reproducibility of the work that we publish. This form is intended for publication with all accepted papers reporting claims of lasing and provides structure for consistency and transparency in reporting. Some list items might not apply to an individual manuscript, but all fields must be completed for clarity.

For further information on Nature Research policies, including our data availability policy, see Authors & Referees.

Experimental design

Please check: are the following details reported in the manuscript?

1. Threshold

Plots of device output power versus pump power over a wide range of values indicating a clear threshold

☐ Yes
☒ No

It is not a pump-probe study.

2. Linewidth narrowing

Plots of spectral power density for the emission at pump powers below, around, and above the lasing threshold, indicating a clear linewidth narrowing at threshold

☐ Yes
☒ No

It is not a pump-probe study.

Resolution of the spectrometer used to make spectral measurements

☒ Yes
☐ No

Resolution of the spectrometer is included in the Methods section of the Manuscript.

3. Coherent emission

Measurements of the coherence and/or polarization of the emission

☒ Yes
☐ No

Polarization information can be found in the Methods section of the Manuscript.

4. Beam spatial profile

Image and/or measurement of the spatial shape and profile of the emission, showing a well-defined beam above threshold

☒ Yes
☐ No

We filter the beam spatially. Thus the profile is Gaussian (see SI).

5. Operating conditions

Description of the laser and pumping conditions
Continuous-wave, pulsed, temperature of operation

☒ Yes
☐ No

The laser conditions can be found in the Methods section of the Manuscript.

Threshold values provided as density values (e.g. W cm^{-2} or J cm^{-2}) taking into account the area of the device

☒ Yes
☐ No

This can be found in the Methods section of the Manuscript.

6. Alternative explanations

Reasoning as to why alternative explanations have been ruled out as responsible for the emission characteristics
e.g. amplified spontaneous, directional scattering; modification of fluorescence spectrum by the cavity

☒ Yes
☐ No

The resonance studies show that we measure inelastic scattering (see SI).

7. Theoretical analysis

Theoretical analysis that ensures that the experimental values measured are realistic and reasonable
e.g. laser threshold, linewidth, cavity gain-loss, efficiency

☐ Yes
☒ No

Not applicable.

8. Statistics

Number of devices fabricated and tested

☐ Yes
☒ No

Not applicable.

Statistical analysis of the device performance and lifetime (time to failure)

☐ Yes
☒ No

The study focuses on single crystals rather than devices, but we looked typically at several samples.

March 2018
